# Systolic Blood Pressure Time in the Target Range and Blood Pressure Variability: The Effects of Amlodipine‐Based Therapy

**DOI:** 10.1111/jch.70160

**Published:** 2025-10-17

**Authors:** Longguo Zhao, Vipin Kumar, Megumi Narisawa, Yanglong Li, Chunzi Jin, Xian Wu Cheng

**Affiliations:** ^1^ Jilin Provincial Key Laboratory of Stress and Cardiovascular Disease Department of Cardiology and Hypertension Yanbian University Hospital Yanji Jilin P.R. China; ^2^ Department of Cardiology Nagoya University Graduate School of Medicine Nagoya Aichi Japan; ^3^ Department of Radiology Yanbian University Hospital Yanji Jilin P.R. China

**Keywords:** blood pressure, variability, calcium channel blocker, pressure lowering

The study by Dr. Yang et al. [1] in this issue of *The Journal of Clinical Hypertension* contributes to the understanding of how individuals in different age groups respond to amlodipine‐based therapy for primary hypertension, which affects nearly 1.3 billion people worldwide and is the leading modifiable risk factor for cardiovascular morbidity and mortality [2]. The accurate diagnosis of hypertension is challenged by the inherent variability of blood pressure (BP) measurements, since BP naturally fluctuates and is influenced by circadian rhythms and various environmental and physiological factors [3]. The variability in BP values complicates hypertension diagnoses and can result in misclassification when measured at a single time; this is further complicated by white‐coat hypertension and masked hypertension [4]. Long‐term BP variability (BPV) has emerged as an independent predictor of cardiovascular outcomes, providing additional prognostic information beyond that of the mean BP. BPV is associated with an increased risk of cardiovascular events in patients with hypertension, regardless of their baseline cardiovascular risk [5].

The systolic BP time in the target range (TTR) discussed by Yang et al. in their study was introduced as a comprehensive metric for evaluating long‐term hypertension management. The TTR integrates both the mean BP level and BPV, offering a more robust assessment of BP control over extended periods. BP in the TTR is negatively associated with mortality, cardiovascular disease, and kidney complications in hypertensive patients [6, 7]. Amlodipine, a widely prescribed calcium channel blocker (CCB), has received particular attention among antihypertensive agents for its potential to optimize these newer metrics (Figure 1). Treatment with amlodipine provides sustained antihypertensive effects and has been shown to reduce BPV more effectively than other CCBs in clinical settings [8].

**FIGURE 1:**
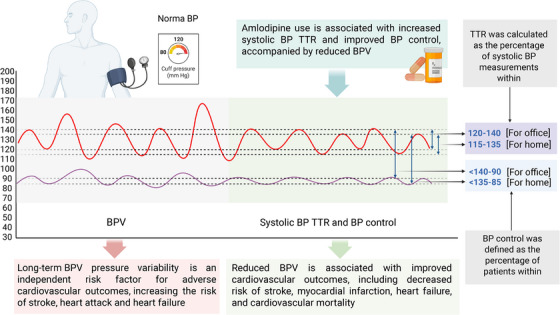
Effect of amlodipine on systolic BP TTR and BPV. BP, blood pressure; BPV, blood pressure variability; TTR, time in target range.

Yang et al.’s retrospective cohort study encompassing >36 000 patients in the China Hypertension Center database provides valuable insights into the effectiveness of amlodipine treatment across different age groups, with particular emphasis on novel measures including the TTR and BPV. Their study's focus on the TTR and BPV reflects the increasing recognition that these measures may be as important as mean BP readings. The TTR and BPV have gained importance due to evidence that visit‐to‐visit BPV may be as significant as the mean BP level in predicting cardiovascular outcomes, as demonstrated in landmark studies such as the Anglo‐Scandinavian Cardiac Outcomes Trial (ASCOT) [9]. That trial included patients receiving amlodipine‐based therapy (including amlodipine monotherapy or combination therapy) at baseline and during the follow‐up, representing a clinically relevant population and reflecting real‐world therapeutic approaches. The study's four‐group age stratification (18–45, 46–64, 65–79, and ≥80 years) was well‐conceived, capturing distinct physiological and clinical phases of hypertension management.

Yang et al. provide comprehensive outcome definitions and detailed calculation methods. The TTR calculation uses a weighted approach that considers the percentage of systolic BP measurements within the therapeutic range, weighted by the time interval between visits. The target ranges of 120–140 mmHg for office measurements and 115–135 mmHg for home measurements align with contemporary guidelines and acknowledge the established difference between office and home BP readings. The BPV assessment using the coefficient of variation (the systolic BP standard deviation/mean systolic BP × 100%) applied by Yang et al. is a standardized approach that allows for meaningful comparisons across different baseline BP levels. The BP control definition used in their study (<140/90 mmHg in the office or <135/85 mmHg at home) follows established guidelines and provides a clinically relevant endpoint. The average annual rate of increase (AARI) was calculated using the following formula: AARI = [(an/am) ^(1/(n‐m)) − 1], where “am” is the initial value, “an” is the final value, and “n‐m” is the number of yearly intervals between observations. This formula provides a standardized and useful measure of the annualized change rate between the first and last available data points.

The study by Yang and colleagues demonstrated age‐related patterns across various parameters. Their subjects' systolic BP TTR progressively decreased with advancing age, declining from 82.52% in the youngest group to 78.33% in the oldest group (*p* < 0.001). The BP control rates also showed a negative correlation with age, declining from 84.04% in the youngest group to 79.59% in the oldest group (*p* < 0.001). Yang et al.’s analyses also revealed interesting BPV patterns: cross‐sectionally, there was an age‐related increase in BPV, although this trend did not reach statistical significance. The increase from 4.90% in the youngest group to 5.13% in the oldest group aligns with pathophysiological changes that are known to be associated with aging, including arterial stiffening and reduced baroreceptor sensitivity.

However, longitudinally, all of the age groups in the study by Yang et al. showed improvement in BPV over time, with notable annual reductions observed across the groups. During the follow‐up period, the AARI data showed annual improvements across multiple parameters: the TTR (from 1.89% to 3.66% annually), BPV (−1.49% to −16.71% annually), and BP control (1.50% to 2.41% annually). The pattern of improvement varied by age, with the younger patients showing greater TTR improvement (3.66% annually) while the older patients demonstrated greater BPV improvement (−16.71% annually). These improvements suggest that continued amlodipine therapy provides progressive benefits, supporting long‐term treatment strategies.

The analyses conducted by Dr. Yang et al. also revealed concerning patterns of cardiovascular risk‐factor clustering in the younger hypertensive patients. Their 18–45 age group demonstrated higher body mass index values (26.27 kg/m^2^), increased prevalences of alcohol (32.04%) and tobacco (30.66%) consumption, and metabolic disorders, including hyperuricemia (14.32%) and obstructive sleep apnea syndrome (1.94%). This profile suggests that young‐onset hypertension in China is increasingly associated with lifestyle‐related cardiovascular risk factors. The higher diastolic BP in the study's younger patients (98.18  vs. 80.96 mmHg in the oldest group) is particularly noteworthy, as isolated diastolic hypertension in young adults has been associated with increased long‐term cardiovascular risk.

Despite its strengths, the Yang study has several important limitations. We note that the study's reliance on patients enrolled in the China Hypertension Center may introduce selection bias toward more motivated patients receiving care at specialized centers, potentially limiting the study findings' generalizability to the broader hypertensive population. Another significant limitation is the absence of comparison groups receiving alternative antihypertensive regimens. Although valuable data on outcomes with amlodipine‐based treatment are provided by the Yang et al. study, that investigation cannot address whether these benefits are specific to amlodipine or reflect general patterns of structured long‐term antihypertensive therapy.

In addition, the assessment of BPV based on home BP measurements in the Yang study, while following standardized protocols with professional training provided to all participants, may have introduced measurement variability related to patient measurement technique and device calibration despite these safeguards. We speculate that office‐based BPV assessments and/or ambulatory BP monitoring might provide more standardized and reliable data. Perhaps the most significant limitation of the study by Yang et al. is the absence of hard cardiovascular endpoint data. Although the TTR, BPV, and BP control are important intermediate outcomes, their relationship to cardiovascular events, particularly in different age groups, remains unclear in the study.

Yang et al. examined the effects of amlodipine‐based therapy on the TTR, BPV, and BP control across different age groups, and their findings support amlodipine's unique position among the CCBs for long‐term hypertension management. The study demonstrated that although the hypertensive patients in each of the age groups maintained a TTR above 78% and achieved BP control rates near 80%, age‐related differences existed. Younger patients demonstrated better TTR and control rates, while older patients showed higher baseline BPV, which is consistent with expected physiological changes. The study thus provides useful real‐world evidence on age‐specific patterns with amlodipine therapy in hypertensive patients and highlights the potential value of TTR and BPV as monitoring tools in clinical practice.

## Author Contributions

Longguo Zhao wrote the first draft of the manuscript. Vipin Kumar and Megumi Narisawa drafted figure. Yanglong Li and Chunzi Jin edited the manuscript. X.W. Cheng handled the funding and supervision.

## Conflicts of Interest

The authors declare no potential conflicts of interest with respect to the research, authorship, and/or publication of this manuscript.

## Data Availability

The authors have nothing to report.
